# Effects of proton pump inhibitor versus H2-receptor antagonist stress ulcer prophylaxis on ventilator-associated pneumonia: a pilot study

**DOI:** 10.1186/cc12340

**Published:** 2013-03-19

**Authors:** JF Fogas, KK Kiss, FG Gyura, ZT Tóbiás, ZM Molnár

**Affiliations:** 1 University of Szeged, Hungary

## Introduction

Ventilator-associated pneumonia (VAP) is the most frequent ICU-acquired infection among patients receiving mechanical ventilation with relative risk of 9 to 27% and with mortality of 25 to 50% [1,2]. One of the promoting factors of VAP is the increased pH of the gastric acid, which occurs when H2-receptor antagonists (H2RA) or proton pump inhibitors (PPI) are used for stress ulcer prophylaxis. The aim of this study was to investigate effects of PPI versus H2RA on the occurrence of VAP and, as second endpoint, the occurrence of gastrointestinal bleeding (GIB).

## Methods

After ethics committee approval mechanically ventilated (>48 hours) adults were recruited in a prospective randomized controlled, double-blind clinical trial and randomised into PPI or H2RA groups. Patients with admission diagnosis of pneumonia or other acute inflammatory pulmonary disease, severe sepsis/septic shock, and/or regular PPI/H2RA users were excluded. The diagnostic criteria for VAP were: leukocytosis, increased PCT level, fever, purulent tracheal secretion, positive microbiological result of tracheal sample, and new/ increased infiltrate on chest X-ray. Data are presented as median (interquartile range, IQR). For statistical analysis Mann-Whitney U test and chi-square tests were used.

## Results

Out of 94 patients recruited 14 were excluded due to early weaning/death (*n = *9) or protocol violation (*n = *6). The remaining 79 patients were analysed (PPI, *n = *38; H2RA, *n = *41). There was no significant difference (*P >*0.05) between the groups regarding demographics: age 67 (56 to 77) versus 72 (58 to 79) years; male/female: 23/15 versus 25/16; length of mechanical ventilation: 5 (3 to 9) versus 5 (2 to 8) days; APACHE II score: 28 (22 to 32) versus 26 (21 to 36) (PPI vs. H2RA, respectively). There was no significant difference in the number of cases with VAP in the PPI versus H2RA groups: 9 (24%) versus 10 (24%). None of the patients developed GI bleeding during their stay on the ICU.

## Conclusion

The results of this pilot study suggest that there may be no difference in the incidence of VAP and GI bleeding if stress ulcer prophylaxis is performed by H2RA or PPI. As the latter is more expensive, its use as first choice in critical care should be questioned.

However, the completion of the study on the planned 198 patients is required to come to the final conclusions.
